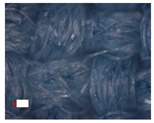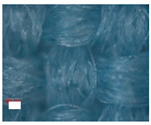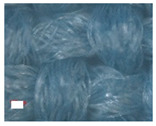# Tailoring of Durable Conductive and UV-Shielding Properties on Cotton and Polyester Fabrics by PEDOT:PSS Screen-Printing

**DOI:** 10.3390/polym12102356

**Published:** 2020-10-14

**Authors:** Alenka Ojstršek, Selestina Gorgieva

**Affiliations:** 1 Faculty of Mechanical Engineering, Institute of Engineering Materials and Design, University of Maribor, Smetanova 17, 2000 Maribor, Slovenia; alenka.ojstrsek@um.si; 2 Faculty of Electrical Engineering and Computer Science, Institute of Automation, University of Maribor, Koroška cesta 46, 2000 Maribor, Slovenia

**Keywords:** cotton, polyester, screen-printing, conductive polymer, wash and wear durability, functional properties

## Abstract

In the present study, cotton (Co) and polyester (PES) fabrics were screen-printed with a conductive poly3,4-ethylenedioxythiophene:polystyrene sulfonate (PEDOT:PSS) printing paste along with a commercially-available screen-printing binder (SFXC) or waterborne polyurethane resin (WPU), in order to enhance wash and wear durability, and to improve some functional properties, without essentially influencing the physical–mechanical properties of the base material, as well as the introduced fabrics’ conductivity. The application of a conductive polymer coating reduced transmittance in the whole UV region drastically, indicating good UV-shielding ability in the treated fabrics. Moreover, the employed binders improved the fabrics’ protection against harmful solar UV radiation significantly, depending on the type of fibre and binder. Furthermore, the SFXC binder intensified the hydrophobicity of Co as compared to the WPU binder, and, on the other hand, WPU reduced the hydrophobicity of PES. Finally, the screen-printed fabrics were washed up to 20 cycles and rubbed up to 20,000 cycles, and characterised by means of mass loss determination and electrical resistivity measurement. Both binders enlarged polymer stability against the effect of washing and rubbing, depending on the number of cycles, the type and amount of employed binder, the type of fibres, and the thickness and uniformity of coatings.

## 1. Introduction

The industrial and scientific community has recently been showing a growing interest in the field of conductive textiles that, in convergence with electronics, is leading to the development of smart or electronic textiles (E-Textiles), which could be used for diverse application fields, e.g., in healthcare, security, entertainment, space exploration, sports and consumer fitness fields, etc. [1,2], with the aim to serve and facilitate our lives. Up to now, numerous approaches have been proposed to obtain conductive textiles, namely, weaving and knitting of conductive yarns into the textile, sewing and embroidering of threads on the surface, electroless metal deposition, incorporation of metal wires in the fabric, coating or printing of conductive polymers and inks on the surface, etc. [3,4,5].

Employment of conductive polymers for the fabrication of flexible and wearable electronics has some advantages over the aforementioned techniques, such as being lightweight, having low processing temperatures, low cost, stretchability, foldability, good adhesion to diverse substrates, and compatibility with various processing techniques [6,7]. Unlike the metals, they do not cause skin irritation and long-term toxicity in direct contact with the skin [8]. Moreover, they can be applied on a larger surface area with potential applications for sensors, thermoelectrics, thermocouples, antennae, wearable electronics, and displays such as organic light-emitting diodes (OLEDs), radio frequency identification tags (RFIDs), electromagnetic shielding, high surface area electrodes for capacitors and/or batteries, etc. [7,8,9], although they have a remarkably lower conductivity compared to, e.g., electroless copper-plated textiles [10].

Among the various types of available organic conductive polymers for the fabrication of conductive textiles, i.e., polypyrrole (PPy), polyaniline (PANI), and poly-3,4-ethylenedioxythiophene (PEDOT), etc., the poly-3,4-ethylenedioxythiophene: poly-styrenesulfonate (PEDOT:PSS) is considered to be one of the most promising candidates for manufacturing purposes, due to its ease of processing, water solubility, long-term stability, and good film-forming properties [11]. Furthermore, the conductivity of PEDOT:PSS could be enhanced by orders of magnitude, if a polar solvent were added to a PEDOT:PSS aqueous solution as the secondary dopant [12]. PEDOT:PSS has been reported to have no cytotoxicity [8].

Since textiles are subjected to processes and activities during their lifetime of use, e.g., washing, drying, and flexing, diverse compounds could be employed to improve the adhesion and thus wash and wear resistance of conductive polymer coatings. Åkerfeldt et al. [13] used a laboratory version of knife-over-roll coating for the application of PEDOT:PSS conductive polymer, together with a thermoplastic polyurethane binder and ethylene glycol. Skrifvars et al. [14] combined PANI or PPy with an acrylic binder, and Weiser et al. [15] combined PANI or PEDOT:PSS dispersions with acrylate, polyurethane or silicone binders. Banaszczyk et al. [16] investigated the long-term high temperature ageing of five different commercially available conductive PPy-coated fabrics potted in epoxy resin. Wu and Hu [17] reported a novel waterborne polyurethane resin (WPU) based thermoelectric composite made of nonionic WPU, multi-walled carbon nanotube (MWCNT), and PEDOT:PSS. Tadesse et al. [3] applied different grades of waterborne polyurethanes in different concentrations to improve the coating and adhesion of the PEDOT:PSS on the fabric. The resistance increased only by a small amount when samples were stretched cyclically at 100% strain, and the samples showed good durability up to 10 washing cycles. However, there is a lack of reported work on the combination of both the improved wear and wash durability of conductive polymer designed to interact with the human body and additional functionalities of the developed coatings.

In the present study, a conductive PEDOT:PSS printing paste was firstly screen-printed on cotton (Co) and polyester (PES) fabrics, with the aim of obtaining sufficient conductivity. In addition, two different binders, i.e., a commercially available screen-printing binder and selected waterborne polyurethane resin, were admixed into printing paste to enlarge polymer stability. Both binders meet specific requirements including reasonable price, wide industrial applicability, low environmental impact, good compatibility with different chemicals, and good adhesion on diverse types of textile materials. The chemical and physical–mechanical properties of the screen-printed samples were characterised and compared with untreated ones, as well as evaluation of the UV-blocking ability and hydrophilic/hydrophobic features of the newly obtained coatings. Samples were further washed up to 20 cycles and rubbed up to 20,000 cycles, and electrical resistivity and mass loss were determined, with the aim of assessing the wash and wear resistivities of the polymer deposit.

## 2. Materials and Methods

### 2.1. Materials

Two industrially bleached fabrics, i.e., plain-weave 100% polyester fabric (PES) with a mass/unit area of 165.0 ± 1.0 g/m^2^ and a thickness of 0.51 mm, warp density of 21 threads/cm and weft density of 19 threads/cm, and plain-weave 100% cotton fabric (Co) with a mass/unit area of 92.7 ± 0.6 g/m^2^ and a thickness of 0.18 mm, warp density of 51 threads/cm and weft density of 44 threads/cm, were used as the substrate materials. In order to remove the dirt from the surfaces, both fabrics were washed at 40 °C for 30 min using a non-ionic detergent, and additionally rinsed in warm and then cold water and dried at an ambient temperature.

Two compounds, SFXC^®^ water-based screen-printing binder (Good Life Innovations Ltd., Special FX Creative, Newhaven, UK) and Elpeguard SL 1305 AQ-ECO (Lackwerke Peters GmbH&Co, Kempen, Germany), and waterborne polyurethane resin (WPU) were employed as binders for a high-conductivity grade blue screen-printing paste Clevios S V4 (Heraeus, Hanau, Germany), a poly3,4-ethylenedioxythiophene: polystyrene sulfonate (PEDOT:PSS) dispersion in glycols, with viscosity of 15-60 dPa∙s. Selected WPU is commonly used as conformal coating for the protection of assembled printed circuit boards or flat packs in diverse industrial sectors, and SFXC is a commonly used commercially-available binder for screen-printing (undisclosed composition). PEDOT:PSS is further abbreviated as P in all Tables and Figures to simplify the designation of the samples.

### 2.2. Preparation of Conductive Fabrics

PEDOT:PSS was admixed with an individual binder, SFXC or WPU, in three different ratios—conductive polymer: binder 9:1, 8:2, and 7:3, using a mechanical stirrer for 30 min to obtain a homogeneous printing paste. Prepared mixtures, as well as pure PEDOT:PSS, were applied onto Co and PES fabrics by means of a screen-printing process, using a semiautomatic printing table Mini-MD Johannes Zimmer, Austria. To achieve uniform coatings over the entire fabric surface, a 125 PES mesh was used with pre-optimised operational parameters, i.e., a roll-rod diameter of 15 mm, speed of 1 m/min, max magnetic pressure and two application layers. After screen-printing, all samples were dried at room temperature for 24 h. In addition, WPU-treated samples were cured at 100 °C for 20 min and SFXC-treated at 165 °C for 2 min, as recommended by the chemicals’ suppliers. In addition, the add-on percentage of the individual printing paste on the surface of Co and PES fabrics was calculated according to Equation (1):

(1) $$\mathrm{Add}-\mathrm{on}\;\left(\%\right)\;=\;\frac{m_{c\;}-\;m_{i}}{m_{i}}\;\times 100$$

where *m_c_* is a mass of screen-printed sample; and *m_i_* is a mass of sample before screen-printing.

Moreover, the relative colour strength (K/S) of applied coatings was calculated at a wavelength of minimal reflectance value (in this case at 690 nm), using the Kubelka–Munk Equation (2):

(2) $$K/S\;=\;\frac{{\left(1-R\right)}^{2}}{2\times R}$$

where K is the absorption coefficient; S is the light-scattering coefficient; and *R* is the decimal fraction of the stained sample’s reflectance. Reflectance was measured by means of a two-rays spectrophotometer Spectraflash SF600 Plus (Datacolor, Lawrenceville, New Jersey, USA) at standard illuminant D65 (LAV/Spec. Incl., d/8, D65/10°).

### 2.3. Washing Durability and Abrasion Resistance

With the aim of evaluating the washing durability of the coatings, screen-printed Co and PES samples were washed in a Labomat (Werner Mathis AG, Oberhasli, Switzerland) up to 20 times, according to Standard ISO 105-C06 at a temperature of 40 °C for 30 min, using a solution of 1 g/L of standard reference detergent without optical brighteners, and a liquor-to-fabric weight ratio of 50:1. After each washing cycle, the samples were rinsed three times under tap water, where each rinsing cycle lasted for 1 min, and then dried at room temperature. Electrical resistivity of the samples was determined before and after a selected set of washing cycles (5, 10, and 20) as described in 2.9.

The Martindale method was applied for evaluation of the abrasion resistance of different coatings on the fabrics’ surfaces, using Standard EN ISO 12947-3. The mass loss of individual sample was determined after each set of rubbing cycles (5000, 10,000, and 20,000), according to Standard ISO 3801, using a Zweigle KG device (Zweigle Textilprüfmaschinen GmbH & Co KG, Reutlingen, Germany). In addition, the electrical resistivity was measured.

### 2.4. Hydrophilic/Hydrophobic Features

Water Contact Angle (WCA) measurement was performed using the sessile drop technique, with the aim of evaluating the influence of employed coatings on the hydrophobic/hydrophilic features of fabrics. An individual sample was placed on a horizontal table attached to a mechanical device on a Goniometer (DataPhysics Instruments GmbH, Filderstadt, Germany). A micro-drop with the volume of 0.3 μL MilliQ water was poured onto the fabric surface. The drop was illuminated by white diffuse light and observed with a tele-microscope. A clear image of the drop was transferred directly through a CCD-camera showing the drop profile. The contact angle was determined from the tangent to the drop at the three-phase contact line. The average WCA values were obtained by measuring the contact angles at three various positions on the samples, and the standard deviations were calculated.

### 2.5. UV-Shielding Ability

The transmittance spectrum of the individual coated sample was recorded over the UV–vis spectral range of 200–700 nm wavelengths (200–280 nm is UV-C, 280–315 nm is UV-B, 315–400 nm is UV-A, and 400–700 nm is the Vis region) by means of a Lambda 900 UV–vis-NIR spectrophotometer (Perkin Elmer, Waltham, MA, USA) with an integrated sphere, at a scanning speed of 450 nm per min and a resolution of 5 nm. In addition, the UV-shielding capability was expressed as Ultraviolet Protective Factor (UPF), which was calculated according to the following Equation (3) [18]:

(3) $$\mathrm{UPF}=\frac{\sum_{\lambda =290}^{400}E_{\lambda }S_{\lambda }\Delta \lambda }{\sum_{\lambda =290}^{400}E_{\lambda }S_{\lambda }T_{\lambda }\Delta \lambda }$$

where *E_λ_* is a CIE relative erythemal spectral effectiveness; *S_λ_* is a solar spectral irradiance; *T_λ_* is a spectral transmittance of the fabric; Δ*λ* is a wavelength step in nm; and *λ* is a wavelength in nm.

### 2.6. Mechanical Properties

Before testing, all samples were exposed to a standard atmosphere for 24 h in a climatic chamber, according to ISO/R 139 at temperature of 20 ± 2 °C and relative humidity of 65 ± 5 °C.

Selected mechanical properties, such as elongation at break, tensile strength, and the breaking tenacity of untreated and screen-printed samples, were determined according to Standard ISO 13934-1 using a Textechno statigraph M test machine (Textechno H. Stein GmbH & Co. KG, Moenchengladbach, Germany). A total of 5 measurements were taken separately in both weft and warp directions for each sample (size of 25 cm × 5 cm), in order to obtain statistically significant results.

### 2.7. Optical Microscopy (OM)

The surface appearance of samples was observed by an Axiotech 25 HD (+pol) Zeiss optical microscope (Carl Zeiss NTS GmbH, Oberkochen, Germany), equipped with an Axiocam MRc (D) high-resolution camera. All images were taken in light transmission mode, with a halogen light as the light source using 20× magnification.

### 2.8. Fourier Transform Infrared Spectroscopy (FTIR)

Fourier Transform InfraRed (FTIR) measurements of reference and diversely screen-printed samples were accomplished using a spectrophotometer FTIR System Spectrum GX (Perkin Elmer, Waltham, MA, US), with a Golden Gate ATR attachment and a diamond crystal. The transmittance spectra were recorded within the range of 4000–500 cm^−1^, applying 32 scans and a resolution of 1 cm^−1^.

### 2.9. Electrical Resistivity

**Figure 1:**
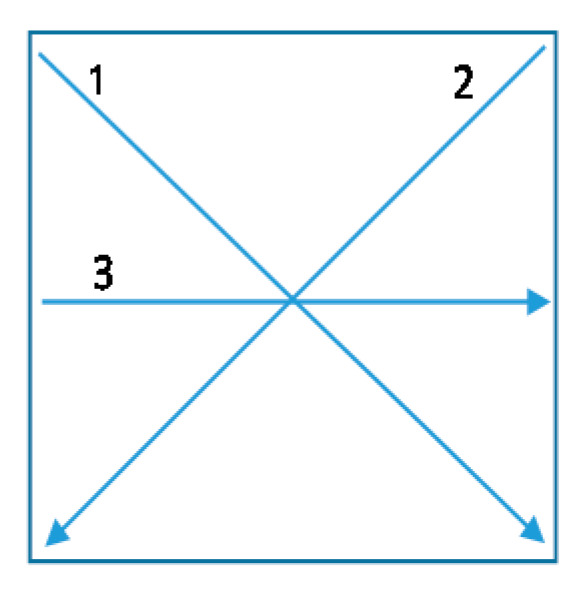
Electrical resistivity’ measuring lines/distances; 1 (5.7 cm), 2 (5.7 cm), and 3 (4 cm).

The electrical resistivity of prepared samples was measured with the use of a set of standardised measuring electrodes at three measuring points/distances on each test sample (Figure 1), using a 34410A 6 ½ Digit Multimeter (Agilent Technologies, Santa Clara, CA, USA).

## 3. Results and Discussion

### 3.1. Characterisation of Screen-Printed Co and PES Fabrics

Table 1 and Table 2 contain the percentage of applied coatings and the relative colour strength (K/S), calculated according to Equations (1) and (2), respectively, on Co and PES fabrics. In addition, the surface appearance of samples was shown. PEDOT:PSS is abbreviated to P in all Tables and Figures to simplify the labelling of the samples.

The applied conductive polymer is visually bluish (OM images in Table 1 and Table 2), causing the blue colouration of samples. As expected, K/S values were higher for PEDOT:PSS treated PES (Table 2) compared to Co (Table 1), since the add-on values were higher, 14.41% (PES) and 10.04% (Co), although those two variables were not directly correlated. Moreover, the higher the binder content, the lower the colour strength, i.e., for Co up to 43.1% (SFXC) and 28.8% (WPU), and for PES up to 28.0% (SFXC) and 25.6% (WPU), as compared to the reference sample. From the OM images in Table 1 it could be clearly perceived that PEDOT:PSS was screen-printed equally on the whole surface of Co, irrespective of paste content. On the other hand, some undesirable aggregation of PEDOT:PSS could be noticed for PES samples—dark blue spots (Table 2), which could lead to non-uniform conductivity and de-adhesion during, e.g., washing, as explained by Tadesse et al. [3]. The (non)uniformity was confirmed by measuring the electrical resistances on three different lines/distances inside individual samples. Furthermore, the electrical resistivities between samples were in the range from 0.27 up to 0.48 kΩ (Co) and from 0.09 up to 0.42 kΩ (PES), which is remarkably lower in comparison with the resistances of applied metals, e.g., electroless copper plated textiles [10], although enough to supply power to LED, also after 20 washing cycles (results in Section 3.3.1). Binders had a negative impact on electrical resistance, SFXC larger than WPU, depending on the polymer: binder ratio.

#### 3.1.1. Mechanical Properties

**Figure 2:**
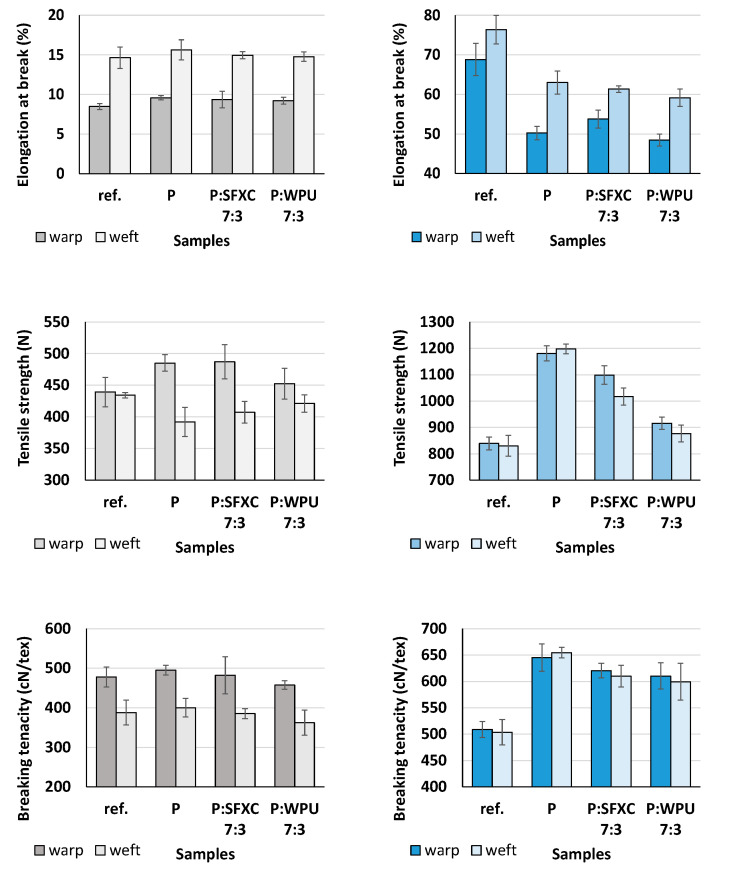
Mechanical properties of selected cotton (Co) (left column) and polyester (PES) (right column) samples in warp and weft directions.

Selected mechanical properties (elongation at break, tensile strength, and breaking tenacity) of selected screen-printed Co and PES (P and P:binder 7:3) were compared to the untreated reference (ref.) samples in both warp and weft directions, in order to estimate if the employed printing pastes had any unfavourable effect on mechanical performance. The gained results are presented in Figure 2.

From Figure 2 it can be perceived that the un-treated reference Co fabric (above) had significantly lower elongation at break and tensile strength in both warp and weft directions, as compared with PES (below), although the difference in breaking tenacity between fabrics was negligible. The application of conductive polymer enlarged elongation at break and breaking tenacity of Co slightly, i.e., from 8.84 up to 9.58 (warp), from 14.64 up to 15.63 (weft), and from 477 up to 495 (warp), from 388 up to 400 (weft), respectively, and, on the other hand, reduced elongation at break of PES significantly, as well as enlarging tensile strength and breaking tenacity of PES drastically. Printing paste penetrates the fabric structure and prevents the yarns’ mobility, causing fabric’s rigidity and inflexibility [19], which is also connected with the composition of the paste, the type of fibres, and undesired linkages between treated fibres [4]. The admixture of binder into conductive paste influenced the mechanical properties of PES more negatively than Co. The loss of tensile strength of screen-printed Co in the weft direction could be attributed to the partial hydrolysis of the cellulose backbone due to the highly acidic nature of PEDOT:PSS (pH < 2.0) as explained by Zahid et al. [20].

#### 3.1.2. Chemical Analysis

**Figure 3:**
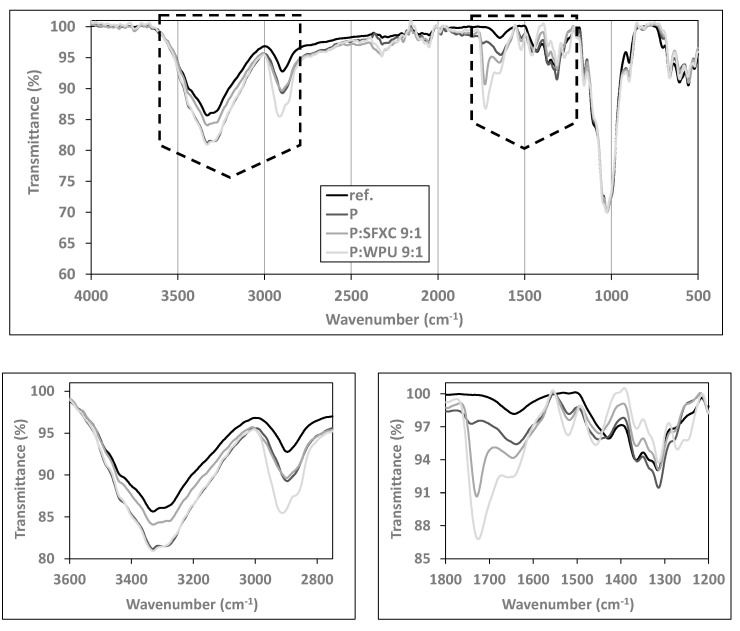
Normalized Fourier transform infrared spectroscopy (FTIR) spectra (at 1850 cm^−1^) of selected Co samples.

**Figure 4:**
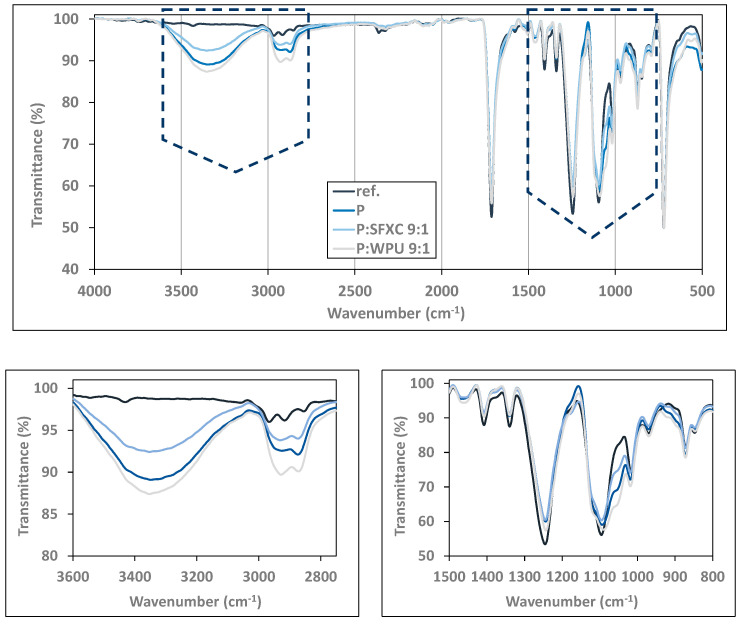
Normalized FTIR spectra (at 1850 cm^−1^) of selected PES samples.

The chemical composition of fabrics’ and respective coatings was identified by recording the FTIR spectra of selected diversely screen-printed Co (Figure 3) and PES (Figure 4). To compare the transmittance intensities of some characteristic peaks, all spectra were normalised at a chosen wavenumber of 1850 cm^−1^, which remained unaffected during surface modification.

From Figure 3 several typical FTIR patterns can be observed for cellulose structure on all inspected samples, irrespective of surface modification, including the stretching absorption of free hydroxyl groups at 3450–3200 cm^−1^, C–H stretching vibrations at 2890 cm^−1^, water molecules at ~1645 cm^−1^, C–H bending vibrations within the glucose ring at 1426, 1369 and 1315 cm^−1^, asymmetric stretching of C–O–C at 1152 cm^−1^, and C–O stretching vibrations at 1056 and 1028 cm^−1^, as also interpreted fully in Ojstršek et al. [21] Figure 4 depicts a FTIR pattern with typical peak positions for PES, including a stretching vibration band of the ester carbonyl group in conjugation with an aromatic ring at 1716 cm^−1^, asymmetric C–C–O vibrations at 1242 cm^−1^, and aromatic C–H wagging at 719 cm^−1^ [22]. Additionally, some characteristic transmittance bands of PEDOT:PSS are evidently recognised for screen-printed samples (more visible at Co—Figure 3): i.e., peaks at 1520 cm^−1^ (C=C), 1315 cm^−1^ (C–C), 920 cm^−1^ (S–O), and 630 cm^−1^ (C–S) corresponded to the vibrations of the thiophene ring in PEDOT [23]. The vibrations at 1180, 1115, and 1056 cm^−1^ on Figure 4 (PES) corresponded to the C–O–C stretching vibrations of the ethylenedioxy group. Moreover, the stretching absorption of free hydroxyl groups at 3450–3200 cm^−1^ and water molecules at ~1645 cm^−1^ were recognised at PES modified samples, revealing more hydrophilic behaviour as compared to pure PES, which agrees with the results obtained by WCA analysis. In addition, the major vibrations at 1725 cm^−1^ were attributed to the carbonyl C=O band of the WPU binder, vibrations at 2917 and 2865 to symmetric and non-symmetric stretching of the C–H bond with carbonyl, and vibrations at 1608, 1570, and 1518 cm^−1^ to N–H bending and C–H stretching of polymerised urethanes [24].

### 3.2. Functional Assessment of Fabrics

#### 3.2.1. Hydrophilic/Hydrophobic Features

**Figure 5:**
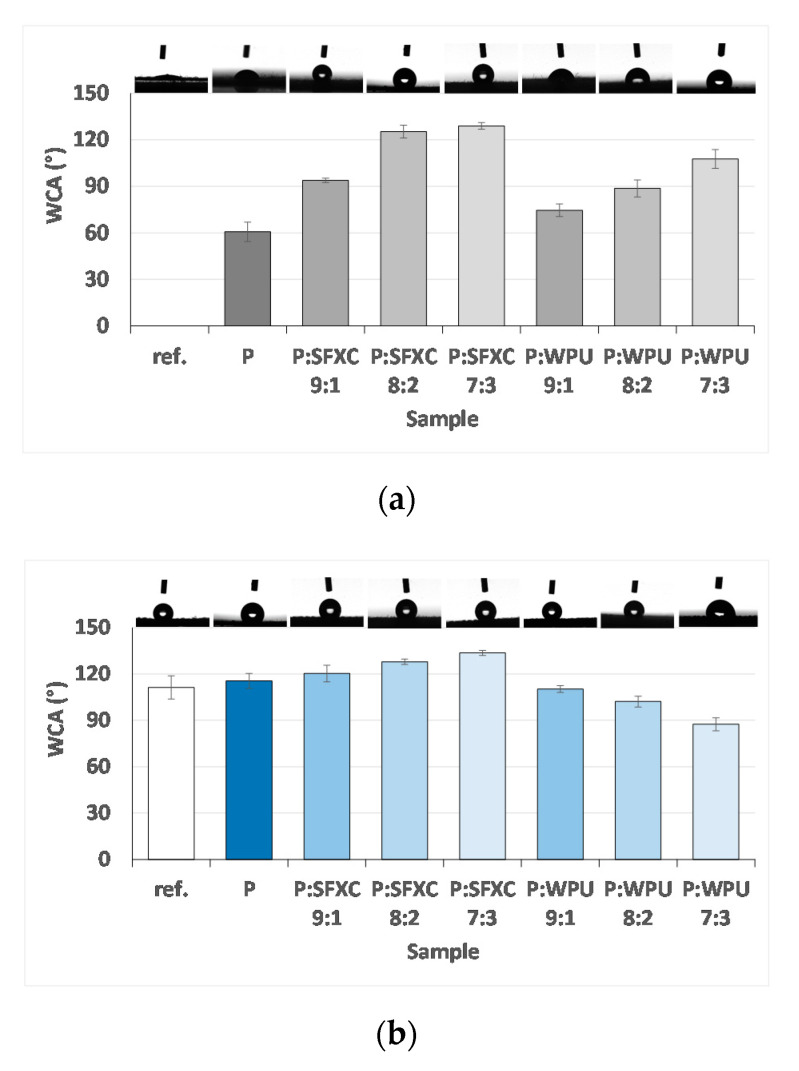
Water contact angle (WCA) of reference and screen-printed samples: (**a**) Co and (**b**) PES.

With the aim of elucidating the role of conductive polymer screen-printed without or together with individual binder on the hydrophilic/hydrophobic features of Co and PES fabrics, coated samples were evaluated via the sessile drop technique using a goniometer set-up, from which the WCAs were determined, and are presented graphically in Figure 5.

PES is an exceedingly hydrophobic (non-polar) synthetic fibre in character [25] compared to the hydrophilic nature of Co, as can also be perceived from Figure 5, where the WCA of un-treated PES was 111.3° ± 7.5° and the WCA of un-treated Co could not be determined. Conductive polymer screen-printed on Co had a temporarily enlarged WCA up to 60° ± 6.2° on average, but after 1 min the water drop slipped completely into the Co surface. In addition, it can be recognised clearly that the SFXC binder intensified the WCA significantly (up to 129° ± 2.2°) in comparison with WPU (107.7° ± 2.2°), depending on the SFXC content, although the enhancement was not linear. A bit of a different situation, with less variation in WCA values, could be perceived for PES fabric. Herein, PEDOT:PSS also changed WCA slightly, when it was combined with the SFXC binder, as in the case of Co. On the other hand, the inclusion of a WPU binder reduced WCAs to 110.3° ± 2.2°, 102.2° ± 3.5°, or 87.5° ± 4.2°, depending on the P:WPU ratio. This could be explained by the fact that, compared to conventional hydrophobic solvent-based polyurethanes, WPUs contain ionic groups and/or non-ionic hydrophilic segments (as perceived from the FTIR results in Figure 4) to disperse them in water [26]. Presumably, the hydrophilic groups of WPU on Co are oriented towards the fibres and hydrophobic outwards, leading to an increase of WCA, depending on WPU content. Just the opposite orientation of WPU is expected on PES, decreasing the WCAs [27].

#### 3.2.2. UV-Blocking Ability of Coatings

**Figure 6:**
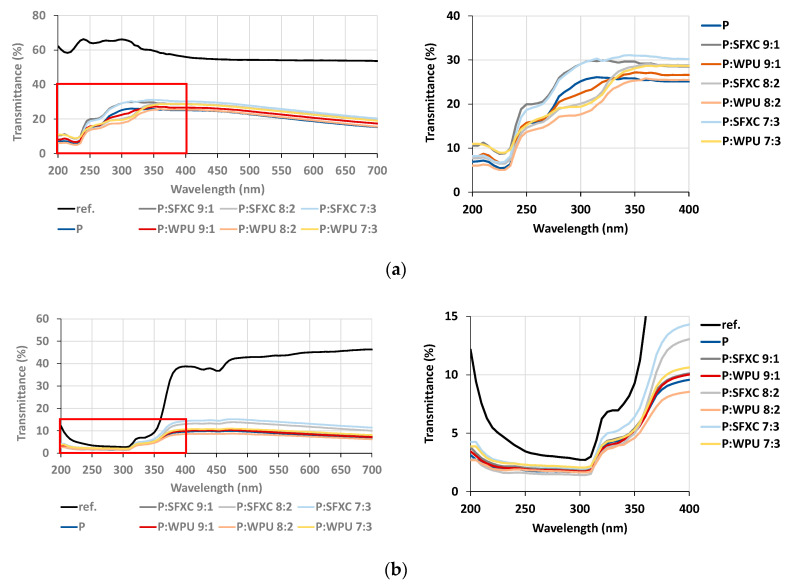
UV–vis transmittance spectra of screen-printed samples and respective references (ref.): (**a**) Co and (**b**) PES. For better visualization, the UV region of interest (red square) is enlarged (right).

The transmittance spectra of Co and PES samples in the UV and vis regions from wavelengths of 200 up to 700 nm are depicted and compared in Figure 6, in order to study the influence of screen-printing paste composition on fabrics’ UV-blocking functionalities. In addition, the UPFs were calculated according to Equation (3).

It is evident from Figure 6a that the pure Co has higher transparency in both UV (60% in average) and vis (54%) regions compared to pure PES on Figure 6b (on average 3% in UV-A, 20% in UV-B, and 44% in vis). The light transmittance through the un-finished fabric depends preferentially on the type of fibres, the fabric’s construction, and pre-treatment process [28]. Accordingly, the calculated UPF of un-treated Co was extremely low, i.e., 3.2 (a non-rateable UV protection level), and UPF for PES fabrics was 21.3, which is in agreement with the results obtained by previous work [21,22,23,24,25,26,27,28]. The application of PEDOT:PSS reduced the transparency in the vis region significantly to 21% (Co) and to 9% (PES) at a wavelength of 550 nm, as expected on account of the thiophene chromophores in a conjugated (PEDOT) polymer, which are responsible for the blue colour of samples with strong light absorbing tendencies. Moreover, transmittance was reduced drastically in the whole UV region, indicating the polymer’s good UV-shielding ability, as also reported by Tian et al. [29] and Sedighi et al. [30]. Therefore, the calculated UPF values were increased up to 15.8 (Co) and 40.3 (PES); that defines good and excellent protective properties, respectively, according to the standard classification [25]. The usage of different binders had a negligible effect on transmittance curves in the vis region, because of their high transparency and colourlessness. On the other hand, the employed binders influenced the UPF significantly, depending on the type of fibre and binder. The presence of SFXC reduced UPF down to 14.9 (Co) and 32.3 (PES), whilst WPU enlarged UPF up to 20.2 (Co) and 43.2 (PES), implying improved fabric protection against harmful solar UV radiation. However, some extra finishing treatments can improve the UPFs further.

### 3.3. Durability

#### 3.3.1. Washing Durability

**Figure 7:**
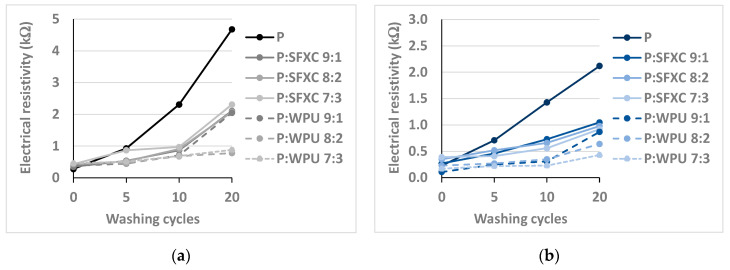

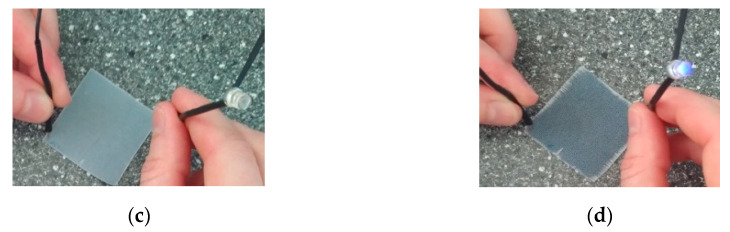
Electrical resistivity versus washing cycles: (**a**) Co and (**b**) PES. Demonstration of screen-printed fabrics for the use in circuits supplying power to LED after 20 washing cycles: (**c**) Co and (**d**) PES.

As employed conductive polymer designed for e-textile applications should be able to withstand severe washing environments, together with intensive mechanical forces. Therefore, samples were washed up to 20 times, and, after several sets of washing cycles (5, 10 and 20), the electrical resistivity was measured between two measuring points at three measuring lines/distances (1, 2, 3) on each sample, as stated in the methodology (Figure 1), allowing the assessment of the conductive properties of the whole sample surface. The average values are presented in Figure 7a (Co) and Figure 7b (PES). Figure 7c,d show the employment of 20 times washed PEDOT:PSS screen-printed Co or PES fabric into an electrical circuit, which, besides a resistor (conductive fabric), consists of a 6 V battery as a source of electricity, LED, and wire.

In Figure 7, the electrical resistivity is shown as a function of the screen-printing paste composition, type of fibres, Co (Figure 7a) and PES (Figure 7b), as well as repeated intensive washings (up to 20). The initial electrical resistances of samples were in a range from 0.11 up to 0.46 kΩ. The higher the binder amount in the printing paste (lower PEDOT:PSS amount), the higher the electrical resistivity was, and consecutively, the lower the conductivity was. In addition, a significant increase in surface resistivity was seen after washings, depending on the initial electrical resistivity, number of cycles, and the type and amount of employed binder. A pure PEDOT:PSS screen-printed Co sample without binder addition recorded the highest electrical resistivity after 20 washing cycles (up to 4.68 kΩ), and, therefore, it was not conductive anymore (Figure 7c). On the contrary, the PEDOT:PSS screen-printed PES sample remained conductive after 20 washing cycles (Figure 7d), as well as all other samples, as expected. Both binders glue the conductive polymer firmly onto the fabrics’ surfaces, and, thus, enlarge their stability against the effect of washing, as also explained by Tadesse et al. [3]. However, some differences in electrical resistivity are noticed between the equally screen-printed Co and PES samples, i.e., lower values at PES compared to Co, due to the fact that resistance is affected by the thickness and uniformity of coatings, the type and arrangement of fibres and yarns in textiles, the geometrical dimensions of the sample and its structure [4].

The aforementioned results agree with the results obtained by Guo et al. [8], which demonstrated that the conductivity of printed PEDOT:PSS conductive wires on PET nonwoven decreased to almost 10% of its initial value after three washing cycles (washing in soap and water, and drying at 60 °C for 12 h). In another study, Ryan et al. [31] reported that the conductivity of silk yarn dyed with PEDOT:PSS did not change relevantly after four repeated machine-washing cycles (30 °C, 50 min, spinning at 900 rpm), using a common detergent.

#### 3.3.2. Rubbing Durability

**Figure 8:**
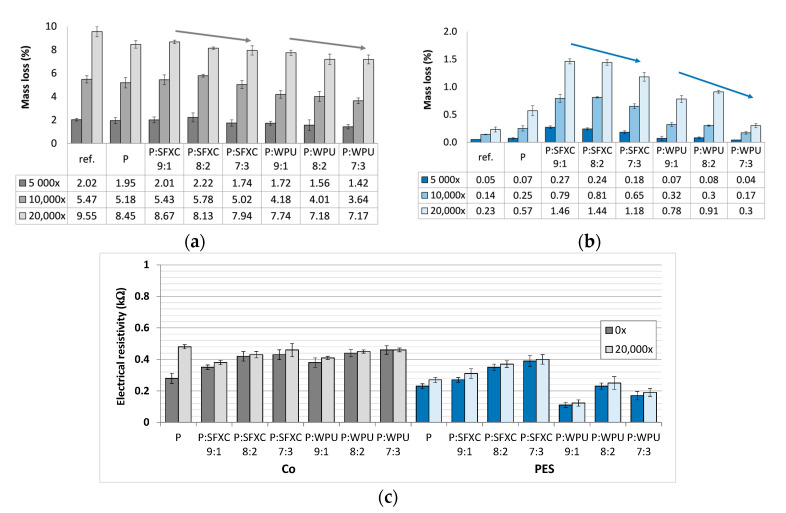
Mass loss of un-treated reference (ref.) and differentially screen-printed samples measured after 5000, 10,000, and 20,000 rubbing cycles: (**a**) Co and (**b**) PES; and (**c**) electrical resistivity of samples after 20,000 rubbing cycles.

When dealing with electrically conductive coatings on textiles, it is significantly important to test their ability to withstand rubbing during wear. Therefore, a standard abrasion resistance Martindale method was employed in the presented research, where an individual sample was exposed up to 20,000 rubbing cycles, and then a mass loss was determined after several sets of rubbing cycles, as well as electrical resistivity after 20,000 cycles (Figure 8).

The untreated Co sample (Figure 8a) had remarkably lower abrasion resistance in comparison to the PES sample (Figure 8b), as expected [32], although the mass loss increased for all (untreated and printed) samples, with an increase of the rubbing cycles, i.e., up to 8.67% (Co) and 1.46% (PES) after 20,000 rubbings. The obtained results of the fabrics’ electrical resistance measured after 20,000 rubbings (Figure 8c) proved that a sufficient quantity of conductive polymer remained on all examined surfaces. As explained by Ojstršek et al. [4], deflection and tension escalates by increasing the rubbing cycles, decreasing the electron mobility within the coating, as also reported by Åkerfield et al. [13], up to the point when filament finally breaks, which did not happen in the present study. The addition of a binder into the printing paste reduced mass loss during rubbing, depending on the type and amount of binder, as well as the type of fibres. Consequently, the electrical resistivities were lower (conductivities were higher) after 20,000 rubbings, as compared to PEDOT:PSS treated samples. In general, coatings increase abrasion resistance since they adhere to the fibres, and thus protect the fabric’s surface [13]. In our case, the WPU binder exhibited better abrasion resistance, as compared to a standard SFXC binder, due to the fact that polyurethane offers an outstanding wear and wash resistance, and is used widely for systems that require high abrasion resistances [33].

## 4. Conclusions

In the present study, Co and PES fabrics were screen-printed successfully using commercially-available highly conductive polymer PEDOT:PSS, alone or together with two selected binders (SFXC or WPU), as confirmed by OM images, FTIR spectroscopic inspection, and electrical resistivity measurement. The application of conductive polymer enlarged elongation at break and breaking tenacity of Co slightly, while it reduced elongation at break significantly and enlarged the tensile strength and breaking tenacity of PES drastically. Admixtures of the selected binders into conductive printing paste influenced the mechanical properties of basic PES more negatively as compared to Co. In addition, PEDOT:PSS was influenced on the functional properties of the base material, by imparting a hydrophobic character to Co fabric, which was further intensified significantly by the addition of the SFXC binder. On the other hand, the addition of WPU in PEDOT:PSS paste reduced the hydrophobicity of PES, due to the presence of ionic groups and/or non-ionic hydrophilic segments. PEDOT:PSS also significantly improved the UV-shielding ability of Co samples in the whole UV region, and somewhat enlarged the UPF of PES. From the results of samples’ electrical resistivities measurements, it could be concluded that both binders glued PEDOT:PSS firmly onto the fabrics’ surfaces, and, thus, enlarged their stability against the effects of washing and rubbing, depending on the number of cycles, the type and amount of employed binder, the type of fibres, and the thickness and uniformity of coatings. By screen-printing of PEDOT:PSS together with a suitable binder, durable conductive and UV-blocking coatings of large surface area can be easily produced, without essentially influencing the physical–mechanical properties of the base material and, thus, the conductive Co and PES fabrics are promising candidates for various wearable e-textiles. The obtained results are part of ongoing project and will be further used in the production of assistive technology for elderly people.

## Figures and Tables

**Table 1:** Add-on percentages, relative colour strength (K/S) values, and optical microscopy (OM) images of screen-printed cotton (Co) samples. Scale bar is 50 μm.

**Sample**	**P**	**P:SFXC 9:1**	**P:SFXC 8:2**	**P:SFXC 7:3**
Add-on (%)	10.04	9.57	9.38	8.48
K/S	1.53	1.18	1.05	0.87
OM	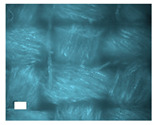	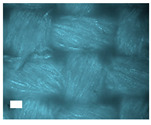	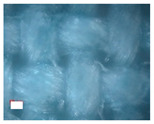	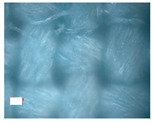
**Sample**	**P:WPU 9:1**	**P:WPU 8:2**	**P:WPU 7:3**	
Add-on (%)	10.07	10.33	10.35	
K/S	1.17	1.14	1.09	
OM	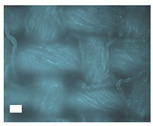	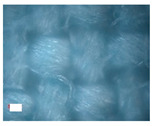	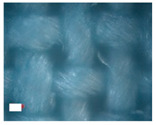	

**Table 2:** Add-on percentages, K/S values, and OM images of screen-printed polyester (PES) samples. Scale bar is 50 μm.

**Sample**	**P**	**P:SFXC 9:1**	**P:SFXC 8:2**	**P:SFXC 7:3**
Add-on (%)	14.41	13.68	13.04	12.95
K/S	1.68	1.49	1.33	1.21
OM	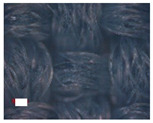	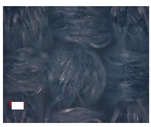	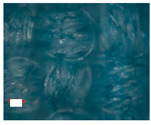	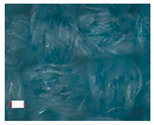
**Sample**	**P:WPU 9:1**	**P:WPU 8:2**	**P:WPU 7:3**	
Add-on (%)	14.21	14.18	14.57	
K/S	1.56	1.31	1.25	
OM